# Control of anterior segment using an antero-posterior lingual sliding retraction system: a preliminary cone-beam CT study

**DOI:** 10.1186/s40510-017-0202-0

**Published:** 2018-01-15

**Authors:** Min Hwang, Hyo-Won Ahn, Soon-Yong Kwon, Jeong-Ho Choi, Seong-Hun Kim, Gerald Nelson

**Affiliations:** 1 Department of Orthodontics, Graduate School, Kyung Hee University, #1 Hoegi-dong, Dongdaemun-gu, Seoul, 130-701 Republic of Korea; 2 Department of Orthodontics, School of Dentistry, Seoul National University, Seoul, South Korea; 3 Division of Orthodontics, Department of Orofacial Science, University of California San Francisco, San Francisco, CA USA

**Keywords:** Lingual orthodontics, Torque, Intrusion, CBCT, Alveolus, TSADs

## Abstract

**Background:**

This study was performed to evaluate the treatment effects of the antero-posterior lingual retractor (APLR), focusing on the 3-dimensional (3D) tooth movement of the maxillary anterior teeth and their alveolar bone levels.

**Methods:**

En masse retraction was performed using either the C-lingual retractor (CLR, C-group, *n* = 9) or the antero-posterior lingual retractor (APLR, AP-group, *n* = 8). We evaluated 3D movement of the maxillary anterior teeth and alveolar bone levels, root length of the central incisors, long axes of the maxillary canines, and occlusal plane changes from CBCT images.

**Results:**

After retraction, the central incisors were more significantly intruded and their root apex was more retracted in the AP-group. The long axis of the canine was well maintained in the AP-group. There were no differences in the steepness of occlusal plane and the incidence of alveolar bone loss or of root resorption during en masse retraction with the two retractors.

**Conclusions:**

The clockwise bowing effect of the anterior segment was less with the APLR, which prevented unwanted canine movement.

## Background

Lingual orthodontic appliances can be classified into either continuous or sectional appliances. The C-lingual retractor (CLR) is a type of sectional appliance, which involves splinting six anterior teeth together and retracting them as a single unit; this method avoids friction between the brackets and archwire and prevents round tripping [1]. In cases of extraction, retraction using a CLR with temporary skeletal anchorage devices (TSADs) has the advantages of esthetically favorable results and early soft tissue change [2, 3].

However, torque control can be more difficult with a CLR, resulting in excess overbite of the anterior teeth and a shallow overbite in the canine region [4]. Recent quantitative studies using cone-beam computed tomography(CBCT) showed that orthodontic treatment with premolar extraction resulted in cortical perforation, root resorption, and bony dehiscence in the lingual area of the upper incisors [5, 6].

**Fig. 1:**
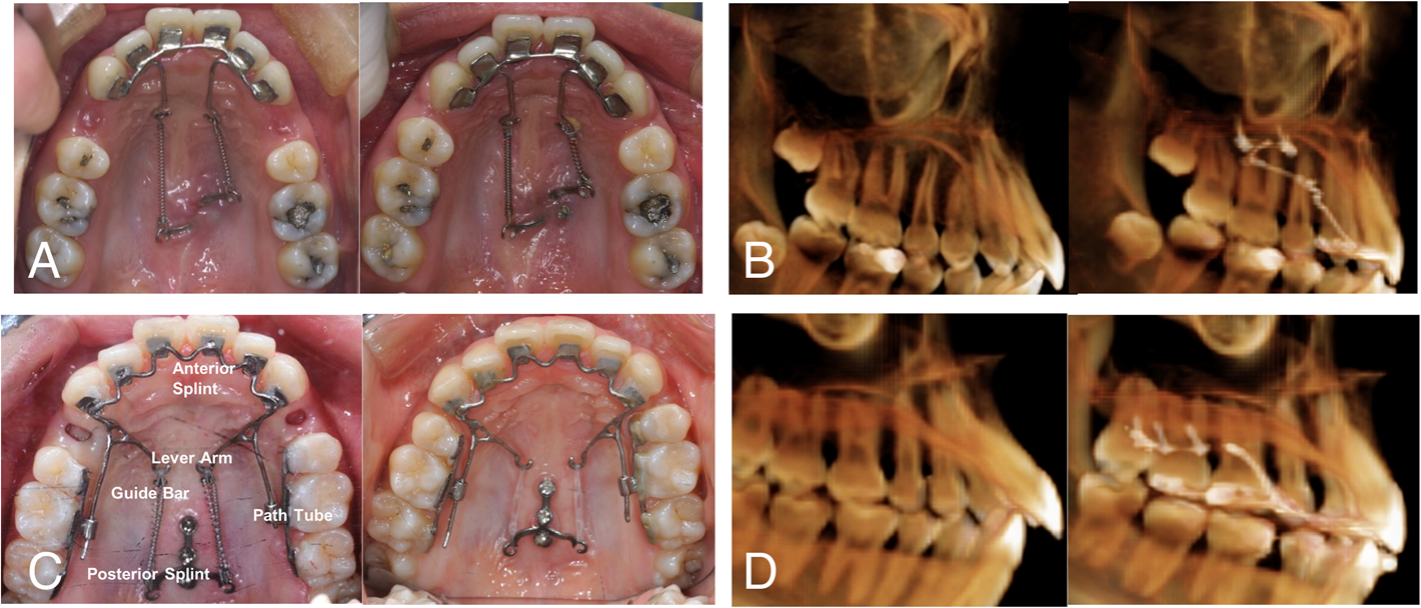
Application of C-lingual retractor (**a** and **b**, CLR) and antero-posterior lingual retractor (**c** and **d**, APLR). Compared to CLRs, APLRs have a guide bar which connects the anterior segment and posterior segment through a path tube. CLR shows the clockwise tipping movement of the anterior segment, whereas APLR shows intrusive retraction with less torque loss from CBCT images

The use of an antero-posterior lingual retractor (APLR) has been proposed to compensate for these limitations of the CLR (Fig. 1a, b) [7, 8]. An APLR consists of a CLR that is attached to the lingual surface of the six maxillary anterior teeth, a splinted segment of the posterior teeth, lever arms, and a tube to create a path for a guide bar. Lever arms are attached to the anterior splinting segment and the path tube is attached to the posterior splinting segment. The 0.036″ guide bar is connected to the middle part of each lever arm and passes through the path tube. The extended guide bar directs the sliding movement in the tube (Fig. 1c, d). In a previous study, the APLR produced a large amount of intrusion and retraction of the anterior teeth with alveolar bone remodeling in hyperdivergent Class II patients, and the alveolar bone volume on the pressure side was preserved [7]. Because this system has directional control, we hypothesized that it would provide improved control over torque and angulation of the anterior segments and prevention of unwanted canine tipping.

The aim of this preliminary study was to compare the treatment effects between C-lingual retractor (CLR) and antero-posterior lingual retractor (APLR) focusing on the 3-dimensional (3D) tooth movement of the maxillary anterior teeth and their alveolar bone level using CBCT analysis.

## Methods

The retrospective data in this study were obtained from 17 patients with anterior protrusion. This study was performed under approval of the Institutional Review Board (IRB, KHD-IRB-1404-2).

The inclusion criteria were as follows (Table 1): (1) ANB 2°–6°, (2) FMA > 25°, (3) nongrowing patients, (4) arch length discrepancy < 3 mm, (5) four first premolar extraction required, and (6) the palatal TSADs were the sole source of anchorage. En masse retraction was performed using either the CLR (C-group *n* = 9; mean age 16.9 years; 8 females, 1 male) or the APLR (AP-group *n* = 8; mean age 20.2 years; 8 females). The period of retraction was 8.4 months (C-group) and 7.8 months (AP-group).

**Table 1:** Cephalometric variables of the samples before treatment

Variables	Control group (*n* = 9)		Experimental group (*n* = 8)		*p* value
	Mean	SD	Mean	SD	
Skeletal					
SNA (°)	80.22	3.69	79.37	3.05	0.615
SNB (°)	76.56	2.79	73.71	4.31	0.122
ANB (°)	3.56	1.96	5.67	2.14	0.051
PFH/AFH	0.62	0.02	0.60	0.03	0.105
SN to OP (°)	20.44	3.87	24.72	3.70	0.035
FMA (°)	29.17	3.90	33.95	6.38	0.078
SN to PP (°)	9.00	3.14	11.65	3.60	0.126
Dental					
IIA (°)	114.50	7.11	122.00	11.39	0.120
FH-U1 (°)	119.00	5.47	108.29	9.15	0.010
IMPA (°)	96.44	6.10	95.76	6.23	0.823
FMIA (°)	50.61	8.72	50.29	8.27	0.939
U1 to NA (mm)	8.67	3.10	6.01	2.14	0.060
U1 to NA (°)	28.89	8.03	18.85	8.12	0.022
L1 to NB (mm)	9.33	2.73	10.84	3.06	0.299
L1 to NB (°)	32.11	7.74	33.48	5.52	0.683
Soft tissue					
UL-E-line (mm)	2.17	1.62	3.39	2.01	0.185

The CLR was soldered to six mesh pads and bonded to the palatal surface of the anterior teeth with a 0.9-mm stainless-steel-wire lever arm (Fig. 1a). The APLR also has 0.9-mm guide bars which extend to pass through the posterior guide tube (Fig. 1c). The guide tube is attached by solder to the posterior splinting assembly that is bonded to the lingual of the posterior teeth or is soldered to the lingual of the molar bands. The C-plate or miniscrews were used as the anchorage unit (Jin-Biomed Co., Bucheon, Korea). After extraction of the premolars, traction between the TSADs and the lingual retractor was applied with an elastomeric chain or NiTi springs, producing a force of 200*g*/side [9].

### CBCT image acquisition and orientation

**Fig. 2:**
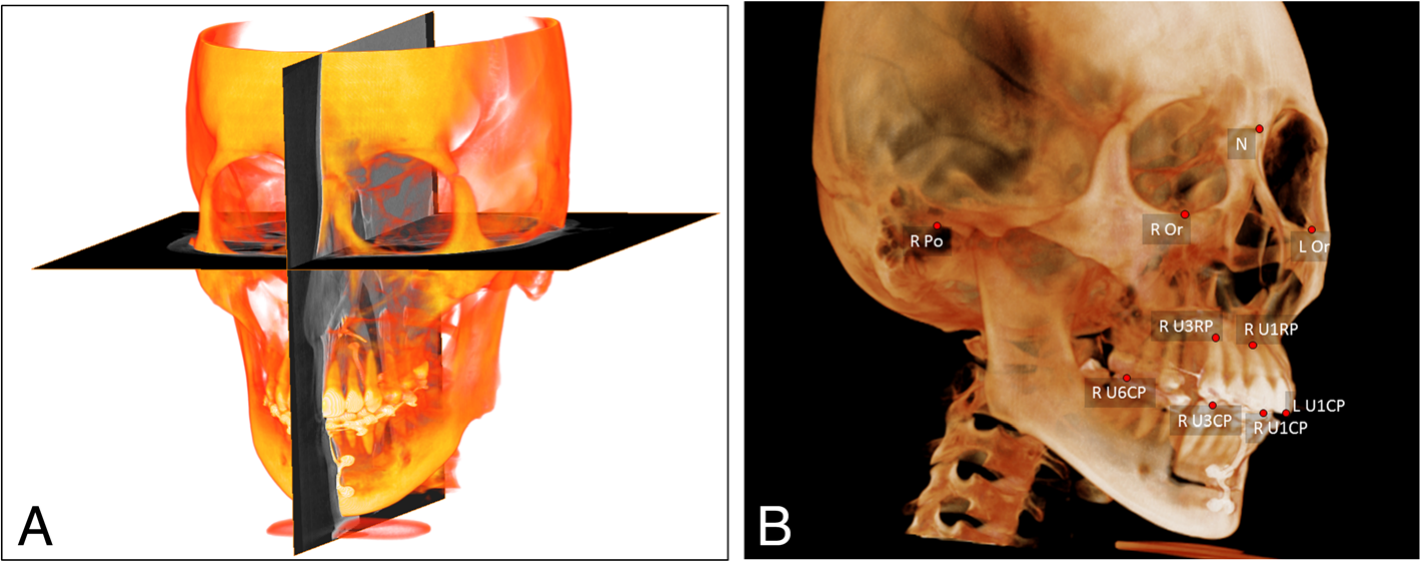
CBCT orientation (**a**) and 3D coordinates of anterior teeth (**b**). **a** The *XY*-plane is parallel to the FH-plane, the *YZ*-plane is parallel to midsagittal plane (perpendicular to FH plane, including Na-Ba line), and origin point is determined to the nasion. **b** U1CP (the maxillary central incisal edge point), U1RP(the maxillary central incisors root apex point), U3CP (the maxillary canine cusp point), U3RP(the maxillary canine root apex point), and U6CP (the maxillary first molar mesiobuccal cusp point) were measured

CBCT images were taken with 0.15 mm^3^ voxel size at the pretreatment (T0) and post-retraction (T1) stages (Alphard-3030; Asahi-Roentgen; Kyoto, Japan) and analyzed by using the InVivoDental (Anatomage; San Jose, CA, USA) and On Demand 3D (CyberMed Inc.; Seoul, Korea) software programs. To set an identical reference point at the T0 and T1 stages, they were superimposed by maximizing mutual information (MI), and the maxillary sinus and palate was designated as the registration area because these anatomic structures do not change during orthodontic treatment. The 3D coordinate point orientation was performed as follows: The *XY*-plane was parallel to the Frankfurt horizontal plane (FH-plane; 3points: both orbitales and the right porion), the *YZ*-plane was parallel to the midsagittal plane (perpendicular to the FH-plane, including the Na-Ba-line), and the point of origin (0,0,0) was determined by the nasion point [10, 11]. In this study, movement of the maxillary teeth was evaluated by comparing the *X*, *Y*, and *Z* coordinates of the tip (CP) and root apex (RP) of the maxillary central incisors and canines and the mesiobuccal cusp of the maxillary first molars (Fig. 2b, Table 1).

### 3D changes of the maxillary anterior teeth, the long axis of the maxillary canine, and the occlusal plane

**Fig. 3:**
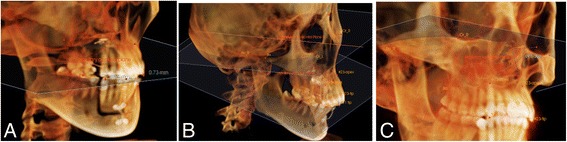
Measurements on occlusal plane and maxillary canine changes. **a** Distance between occlusal plane and the U3CP. **b**. Occlusal plane angle to the FH-plane. **c** Long axis of the maxillary canine relative to the FH-plane

The 3D coordinates were *X*, transverse direction; *Y*, antero-posterior direction; and *Z*, vertical direction. Positive values indicate outward, backward, and upward displacement on the *X*, *Y*, and *Z* planes, respectively. The occlusal plane angle relative to the FH-plane, the long axis of the maxillary canines, and the distance between U3CP and occlusal plane were measured (Fig. 3). The occlusal plane is defined by three points: the cusp tip of the right central incisor and the mesiobuccal cusps of the maxillary first molar on both sides (Fig. 3a) [12].

### Alveolar bone levels and root length (RL) changes of the maxillary incisors

**Fig. 4:**
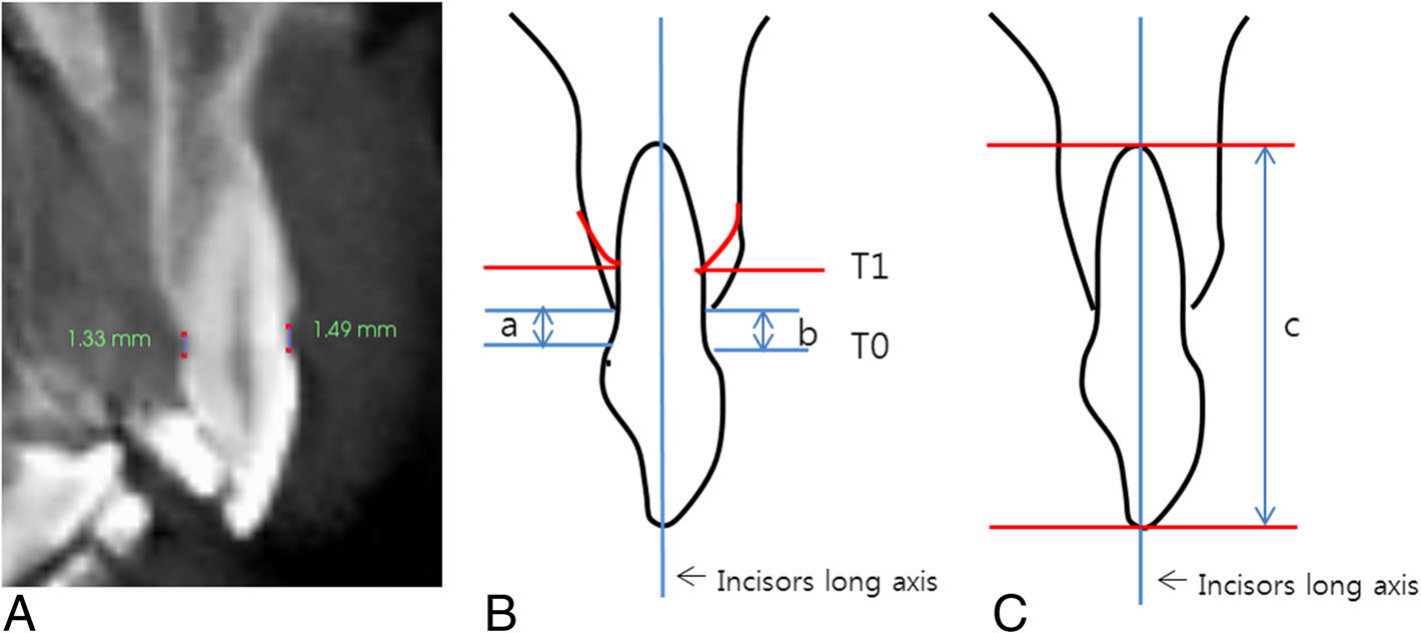
Reorientation of CBCT images for alveolar bone thickness and root length measurement. **a** Reoriented CBCT image. **b** Vertical alveolar bone level on palatal (a) and labial side (b). **c** Root length

The right and left maxillary central incisors and adjacent alveolar bone were measured (Fig 4). The vertical alveolar bone level (VABL) was measured at the labial and palatal sides of the maxillary incisors from the cemento-enamel junction (CEJ) to the alveolar crest (Fig. 4b). The root length (RL) was measured from the incisors tip to the root apex (Fig. 4c) [5, 13].

### Statistical analysis

The normality assumptions of all measured values using Shapiro-Wilk’s test were satisfied. All statistical analyses were performed using SPSS-software (version18.0; SPSS; Chicago, IL). The 3D changes of the maxillary teeth at T0 and T1 were analyzed by paired Student’s *t* tests. An independent *t* test was performed to evaluate the difference between the C-group and AP-group. The level of significance for all of the tests was set at *p* < 0.05.

## Results

### 3D changes of the maxillary anterior teeth, the long axis of the maxillary canine, and the occlusal plane

The central incisors were significantly retracted in both groups (Table 2). The incisal tip of the central incisors was intruded only in the AP-group (U1CPΔZ 1.99 mm, *p* < 0.001). Comparison between groups showed more intrusion of the cusp tips and root apices (*p* < 0.001 and *p* < 0.01, respectively, Table 2) with more retraction of the root apices (*p* < 0.05) of the central incisors in the AP-group. In the canine, there was significant difference between two groups only in the vertical change of the cusp tip (*p* < 0.05; Table 2). The changes in the occlusal plane angle relative to the FH-plane were smaller in the AP-group than in C-group (Table 3).

**Table 2:** Comparison of the changes in CBCT variables between T0 and T1 in each group

Variables	C-group (*n* = 18)				*p* value	AP-group (*n* = 16)				*p* value
	T0	SD	T1	SD		T0	SD	T1	SD	
Three-dimensional tooth movement										
Maxillary central incisors										
U1CP *X*′	4.94	1.78	4.79	1.81	0.383	4.71	1.92	4.58	1.79	0.563
U1CP *Y*	− 9.36	3.51	− 5.34	3.36	0.000***	− 7.64	5.34	− 3.83	4.26	0.000***
U1CP *Z*	− 80.57	2.39	− 80.37	2.45	0.188	− 85.37	4.35	− 83.38	3.87	0.000***
U1RP *X*′	3.20	0.99	3.21	1.29	0.917	3.73	1.83	3.83	1.56	0.393
U1RP *Y*	1.59	3.84	2.53	3.34	0.012*	− 0.47	3.29	1.44	3.36	0.000***
U1RP *Z*	− 60.17	2.23	− 59.21	2.58	0.000***	− 63.37	3.78	− 61.61	3.64	0.000***
Maxillary canines										
U3CP *X*′	17.74	1.85	17.85	1.77	0.510	17.57	2.12	17.91	1.88	0.221
U3CP *Y*	0.65	3.82	4.45	3.99	0.000***	1.50	5.05	5.20	4.48	0.000***
U3CP *Z*	− 79.42	2.13	− 77.95	2.23	0.000***	− 83.53	3.67	− 81.51	3.59	0.000***
U3RP *X*′	13.43	1.77	13.59	1.72	0.270	14.70	1.47	15.14	1.51	0.053
U3RP *Y*	6.59	3.30	7.71	2.63	0.000***	5.48	2.94	6.34	3.22	0.017*
U3RP Z	− 54.77	1.76	− 52.81	1.71	0.000***	− 58.65	3.80	− 56.56	4.17	0.000***
Maxillary first molars										
U6CP *X*′	26.54	1.81	25.76	1.85	0.002**	25.72	2.07	26.72	2.05	0.747
U6CP *Y*	21.54	3.59	20.08	3.12	0.000***	19.88	4.52	19.43	4.30	0.261
U6CP *Z*	− 74.54	2.15	− 75.19	2.14	0.000***	− 79.46	2.83	− 78.47	2.71	0.004**
Alveolar bone level										
VABLl	1.29	0.29	1.36	0.25	0.186	1.70	0.59	1.38	0.61	0.007**
VABLp	1.37	0.28	3.74	2.56	0.001**	1.46	0.47	3.01	1.22	0.000***
Root resorption										
ΔRL	23.62	1.28	22.96	1.59	0.003**	23.55	0.97	22.85	1.30	0.000***

Paired *t* test was performed. The points of #11, 13, and 16 (right sides) were reflected over the *X*-axis (U1CP X′, U3CP X′, U6CP X′, respectively); mirror transformation of *X*-axis. At any point, therefore, outward displacement had a positive value and inward displacement had a negative value

*SD* standard deviation, *Group 1 C-lingual retractor group* C-group, *antero-posterior lingual retractor group* AP-group, *U1CP* the maxillary central incisors cusp tip point, *U1RP* the maxillary central incisors root apex point, *U3CP* the maxillary canines cusp tip point, *U3RP* the maxillary canines root apex point, *U6CP* the maxillary mesiobuccal cusp tip point, *X′* transverse direction (left: +, right: +), *Y* antero-posterior direction (mesial: −, distal: +), *Z* vertical direction (upward: +, downward: −), *VABLl* vertical alveolar bone level on labial side (distance from CEJ to alveolar crest), *VABLp* vertical alveolar bone level on palatal side, *RL* root length (distance from incisor tip to root apex point)

*p < 0.05; **p < 0.01; ***p < 0.001

**Table 3:** Comparison of the changes in CBCT variables between the C-group and AP-group

Variables	C-group (*n* = 18)		AP-group (*n* = 16)		
	Mean	SD	Mean	SD	*p* value
Three-dimensional tooth movement					
Maxillary central incisors					
U1CP Δ*X*′	− 0.16	0.74	− 0.09	0.83	.9291
U1CP Δ*Y*	4.02	1.07	3.81	1.44	.6515
U1CP Δ*Z*	0.20	0.62	1.99	0.69	.0000***
U1RPΔ*X*′	0.02	0.78	0.11	0.46	.7123
U1RP Δ*Y*	0.94	1.41	1.91	1.01	.0364*
U1RP Δ*Z*	0.96	0.93	1.76	0.57	.006**
Maxillary canines					
U3CP Δ*X*′	0.11	0.70	0.34	1.06	.4759
U3CP Δ*Y*	3.80	1.11	3.70	1.23	.7913
U3CP Δ*Z*	1.46	0.71	2.02	0.84	.0436**
U3RP Δ*X*′	0.17	0.62	0.45	0.85	.2801
U3RP Δ*Y*	1.12	1.42	0.87	1.30	.5976
U3RP Δ*Z*	1.97	0.94	2.09	1.58	.7782
Maxillary first molars					
U6CP Δ*X*′	− 0.78	0.93	− 0.09	1.10	.0663
U6CP Δ*Y*	− 1.46	0.90	− 0.45	1.44	.0212*
U6CP Δ*Z*	− 0.65	0.55	0.99	1.05	.0000***
Occlusal plane and canine evaluation					
ΔOP-FH plane (°)	0.62	1.49	− 0.19	2.69	0.446
ΔFH to U3 long axis (°)	5.61	3.23	3.20	2.44	0.020*
ΔOP to U3 cusp tip (mm)	0.15	0.65	− 0.21	0.56	0.093
Alveolar bone level					
ΔVABLl	0.071	0.22	− 0.32	0.42	0.003**
ΔVALBp	2.37	2.51	1.55	1.02	0.783
Root resorption					
ΔRL	− 0.8	0.68	− 0.7	0.59	0.692

Independent *t* test was performed

*SD* standard deviation, *ΔX′* change of *X*-axis (outward movement: +, inward movement: −), *ΔY* change of *Y*-axis (distal movement: +, mesial movement: −), *ΔZ* change of Z-axis (upward movement: +, downward movement: −), *ΔOP-FH plane* occlusal plane angle to FH plane, *ΔFH to U3 long axis* FH plane angle to the long axis of the maxillary canines, *ΔOP to U3 cusp tip* distance between the cusp tip of maxillary canines and occlusal plane, *ΔVABLl* change of vertical alveolar bone level on labial side, *ΔVALBp* change of vertical alveolar bone level on palatal side, *ΔRL* change of root length

*p < 0.05; **p < 0.01; ***p < 0.001

### Alveolar bone level and RL changes of the maxillary central incisors

Between the T0 and T1 stages, the labial alveolar bone levels were either maintained or increased, whereas the palatal alveolar bone levels significantly decreased in both groups (Table 3). Only the change in the labial side of the vertical alveolar bone level was significantly increased in the AP-group compared to C-group (*p* < 0.01, Table 3).

## Discussion

In the present study, we found that use of the APLR compensated for the inherent limitations of anterior sectional retractors, such as the clockwise bowing effect of the anterior segment, canine tipping, and steepening of the occlusal plane [7, 14]. With respect to antero-posterior movement, the APLR induced more bodily movement of the anterior teeth because it had biomechanical properties similar to a continuous arch with a posterior segment (Fig. 1c, d). The guide bar controlled and directed retraction vectors to achieve bodily retraction of the anterior segments. When the anterior teeth were retracted using the CLR, tipping and intrusion of the maxillary canines were observed [14].

With respect to vertical movement, the APLR resulted in full arch intrusion of the maxillary central incisors, canines, and first molars, which resulted in a maintained or flattened occlusal plane. When the intrusive retraction force is applied, the kinetic energy from the guide bar also causes molar intrusion [15]. By contrast, the CLR showed a smaller amount of intrusion of the incisors due to the clockwise vertical bowing of the anterior segment. For treatment of a hyperdivergent patient with a gummy smile, the APLR would be an effective treatment option, requiring palatal TSADs to provide intrusion and retraction to the full maxillary dentition [7].

The main advantage of the APLR is to eliminate the side effects of the CLR and the additional treatment to correct the side effects. This advantage is due to the heavy guide arm that is controlled by the path tube. Another important finding was that favorable alveolar bone response was shown using the lingual retractors, regardless of their types, because they splinted the anterior teeth together. Although the tendency of alveolar bone loss on the palatal side was similar to previous studies [5, 16], the amount of vertical bone loss was much smaller than that of conventional appliances. Ahn et al. [5] also reported that the alveolar bone area increased at the middle level of maxillary incisors on the labial side and decreased in all maxillary incisors on the palatal side.

Recent developments in 3D software programs enable accurate visualization and superimposition of volumes and slices [17, 18]. However, these methods require landmark registration, which can incorporate observer-dependent errors [18]. Kim et al. [19] used this method to obtain geometric information from one software program and then applied it to another, expanding the procedure to include volume and slice-imaging data while refining the algorithm and user interfaces. This method has greatly improved the accuracy of superimposed CBCT data.

Although our sample size was increased by pooling the variables of the right and left sides, it was nonetheless small. In addition, even though both retractors are composed of thick (0.9 mm) stainless steel wires, the lever arm and guide bar could be deflected during retraction, making accurate force application and location of the force vector difficult. Therefore, a modified APLR is proposed, in which the lever arms of both sides are connected to prevent deflection and a trans-palatal arch is added to link the posterior segments [20]. Furthermore, the difference between the angles of the position of the guide bar and tube as well as correlations between tube height and anterior and posterior intrusion should be considered in future studies.

## Conclusions

The study shows that the APLR produced bodily movement and significant intrusion of the anterior teeth was achieved. Some intrusion of posterior teeth was noted. Two retractors did not show different incidence of alveolar bone loss or of root resorption during en masse retraction. The APLR protocol is a good option for the patient who needs intrusion and retraction of the maxillary anterior teeth with good control of the occlusal plane.
